# Immune receptors and aging brain

**DOI:** 10.1042/BSR20222267

**Published:** 2024-02-14

**Authors:** Maja Djurišić

**Affiliations:** Departments of Biology, Neurobiology, and Bio-X, Stanford University, Stanford, CA 94305, U.S.A.

**Keywords:** Complement cascade, Ig-like immune receptors, LilrB2, neuroinflammation, synapse loss, toll-like receptors

## Abstract

Aging brings about a myriad of degenerative processes throughout the body. A decrease in cognitive abilities is one of the hallmark phenotypes of aging, underpinned by neuroinflammation and neurodegeneration occurring in the brain. This review focuses on the role of different immune receptors expressed in cells of the central and peripheral nervous systems. We will discuss how immune receptors in the brain act as sentinels and effectors of the age-dependent shift in ligand composition. Within this ‘old-age-ligand soup,’ some immune receptors contribute directly to excessive synaptic weakening from within the neuronal compartment, while others amplify the damaging inflammatory environment in the brain. Ultimately, chronic inflammation sets up a positive feedback loop that increases the impact of immune ligand–receptor interactions in the brain, leading to permanent synaptic and neuronal loss.

## Immune receptors are nervous system receptors

The involvement of diverse immune receptors in brain ontogeny has been a subject of vigorous investigation for the past two decades. Their role in brain function has been clearly demonstrated from early developmental periods, through healthy adulthood, to aging, as well as pathological changes associated with these life stages [1–4]. Together with the discoveries of a number of other immune-related pathways in the central nervous system (CNS) function, the body of work on immune receptors in the brain contributed to a re-evaluation of the dogma that the brain is an ‘immune-privileged’ organ [5]. This review aims to outline the emerging understanding of how immune receptors in the brain collectively contribute to synapse elimination, neurite dystrophy, and the propagation of inflammation, leading to inflammaging of the brain and ultimately neurodegeneration [6]. For in-depth discussions on specific immune receptors, readers are directed to detailed reviews available elsewhere [7–11].

A number of immune-related pathways are now known to be a part of normal CNS function. These pathways encompass class I major histocompatibility complex (MHC-I) [12,13], class II major histocompatibility complex (MHC-II) [14,15], immunoglobulin-like (Ig-like) [1,12,16,17] receptors, Toll-like receptors (TLRs) [18–20], and various complement components [2,4,21,22]. Although some of these molecules participate in the adaptive immune response, all these pathways are integral to the innate immune response system [10,18,23,24], which in various forms exists since early in evolution [25,26]. In fact, some of the innate immune receptors, like TLRs, are part of evolutionarily primitive neuroimmune cells responsible for both sensing pathogens and mounting defensive response of an early organism [25,27,28]. Given what we now know about various neuroimmune systems in early invertebrates (reviewed in [29]), it is likely that neuro-specific function of various innate immune receptors in mammalian brain reflects their atavistic function. Thus, labeling these molecules as ‘immune,’ derived from their initial discovery in the immune system, might be misleading; these genes exhibit functional pleiotropy, and their roles depend on a set of ligands and downstream signaling cascades specific to the cellular context in which they are found [3,30,31].

Immune receptors are transmembrane molecules that mediate the response of target cells in which they are expressed. The effects of binding of cognate ligands to immune receptors can be grouped into three major categories: (1) triggering of direct cell–cell contacts via ligand-receptor interactions; e.g. T-cells and antigen presenting cells form immunological synapse via interaction between T-cell receptors (TCR) and peptide–MHC complex; (2) promote cytokine or chemokine release by target cells (e.g. binding of pathogen peptides to TLRs in macrophages); (3) control the release of neuropeptides and neurotransmitters [32,33]. In the brain, some receptors function akin to their roles in the immune system, such as initiating cytokine and chemokine release, and phagocytosis [2,9,21]. However, some of the receptors have an unexpected, direct, involvement in regulating synaptic structure and function by virtue of their expression in neuronal compartment [1,16,33,34]. It is also of great consequence that most of the immune receptors have affinity for multiple ligands, which emerge at different times during organismal ontogeny [9,35]. The multi-ligand affinity of these receptors positions them in the brain as sentinels monitoring the changing extracellular milieu, switching their function from normative roles during brain development and healthy adulthood to promoting and reacting to the neuroinflammatory/pathological environment in the aging brain.

## Function of immune receptors in the brain reflects their primordial function

Before the evolution of mammalian immune receptors participating in both innate and adaptive immune systems, immune receptors were part of the environment-sensing mechanism; they bound pathogens but were also deployed in defense as part of primitive integrated neuroimmune systems [29]. The oldest immune receptors in evolution are innate immune pattern-recognition receptors (PRRs), such as TLRs, with extracellular recognition domains that bind stereotypical pathogen-associated molecular patterns (PAMPs) [27]. PRR/TLR-like receptors can be found as far back as Porifera [26,27]; in these early metazoans one of the roles of these receptors is triggering the release of neuropeptides to initiate defense from invading pathogens [26,27,29]. In Hydra, released neuropeptides exhibit a direct antibacterial effect and play a role in shaping its microbiome, which in turn regulates Hydra’s spontaneous contractility [36,37]; this bidirectional interaction is reminiscent of the regulation of the gut–brain axis, including gut motility, in vertebrates by the microbiome [38,39]. Mammalian TLRs function similarly to their counterparts in early metazoans, as they mediate cytokine and chemokine release from immune cells, including microglia in the brain, resulting in the recruitment of other cellular components of the immune system to the site of damage, as well as inflammation that activates adaptive immune system [32,40]. In the brain, TLRs promote microglial phagocytosis [41,42] but also neuroinflammation [43].

Receptors belonging to the immunoglobulin superfamily (IgSF), known as Ig-like receptors, contribute to either innate or adaptive immunity, and some of them have dual function in both immune and nervous system [1,16,17,30,44,45]. In general, Ig-like molecules are characterized by one (e.g. CD3) or multiple copies of Ig-like domains (IgM, TCR, etc.) [46]. Each Ig-like domain consists of an Ig fold, antiparallel β-strands arranged into two sheets linked by a disulfide bond [46]. Despite Ig-like domains forming the basis of adaptive immunity in jawed vertebrates (e.g. MHCs and β2M, TCRs) [47], they are also building blocks of extracellular domains of innate immune receptors (e.g. LILRs), as well as other immune antigens abundant in the brain like Thy-1 [48]. In fact, Ig-fold evolutionarily predates the rearranging receptors of the adaptive immune system, and is frequently deployed for cell–surface interactions [48,49]. The inclination of Ig-like proteins, including MHCs, β2M, and Ig-like domain receptors, for cell–cell and cell–surface interactions is particularly evident in their role in nervous system development, patterning, synaptic formation and pruning, and even pathological protein aggregation [1,13,16,17,30,33,50–57].

The complement cascade, another component of the innate immune system, plays a role in the nervous system. It comprises a canonic set of ligands and receptors recruited in a stereotypical cascade [10]. Complement cascade appeared as early as deuterostome invertebrates and pre-jawed vertebrates, like lamprey [58]. In the CNS, similar to its role in the immune system, the complement cascade is responsible for protecting against invading pathogens [59,60]. However, overlapping mechanisms within the complement cascade are also needed for synaptic pruning during activity-dependent development of thalamic areas [2,21,61,62], as well as increased maturation and migration of progenitors in neurogenic niches of subventricular zone and dentate gyrus [63]. Recent work implicated multiple complement proteins, both early and late in the cascade, as pivotal in promoting neuroinflammation with aging and mediating early synapse loss in Alzheimer’s disease (AD) [64–68].

Numerous genome-wide association studies (GWAS) have linked immune ligands and receptors to aging and age-related neurodegenerative diseases like Alzheimer’s and Parkinson’s [69–72], suggesting their role in etiology or modulation of age-related risk for these diseases. To initiate the discussion, we will first review the role of Ig-like receptors in the aging brain due to their direct involvement in regulating synaptic function and stability [16,33,52]. The role of complement receptors (CRs) and TLRs will be reviewed next in the context of age-related neuroinflammation and neurodegeneration [68,73–76]. A notable characteristic of all three groups of receptors is their affinity for binding misfolded proteins such as amyloid-β and α-synuclein, sometimes with higher affinity than their ‘classical’ ligands [3]. These binding events provide a mechanism for misfolded and aggregated proteins to drive both synaptic loss and inflammation in aging brain [3,9,77].

## Ig-like receptors in neurons promote synaptic elimination in the aging brain

**Figure 1:**
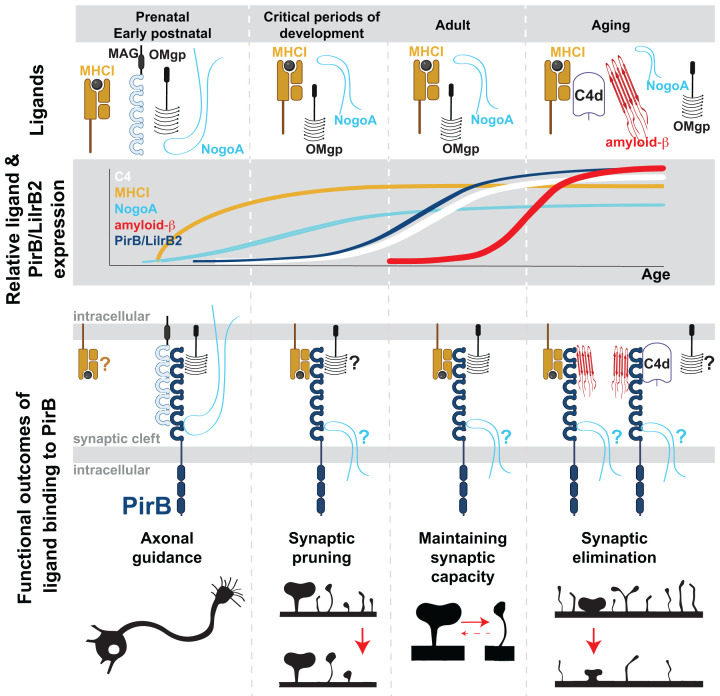
PIRB/LILRB2 receptors’ functional changes across life stages Top panel: Illustration of PIRB/LILRB2 ligands present in the brain during different life stages: prenatal/early postnatal development, critical periods of development, adulthood, and aging. Second panel: Depiction of the relative amounts of the PIRB/LILRB2 receptor and its ligands throughout the four life stages. Third panel: Illustration of different combinations of ligand–receptor pairs and functional outcomes of the binding events for the four life stages. During early brain development: PIRB/LILRB2 binds myelin-associated ligands such as MAG, OMgp, and NOGO-A. Functional Outcome: Negative control of axonal outgrowth towards the target cells. During critical periods of development: PIRB/LILRB2 promotes synaptic pruning via MHC-I ligands. Functional Outcome: Facilitation of synaptic pruning. In adulthood: PIRB/LILRB2 maintains the capacity for plasticity and learning via MHC-I and OMgp. Functional outcome: Support for synaptic plasticity and learning. In aging: PIRB/LILRB2 binds a multitude of ligands with high affinity: oligomeric Aβ, complement proteins, NOGO-A, and viruses (not shown). Functional outcome: Pathological permanent synaptic loss. Expression of LILRB2 receptor and its ligands (MHC-I, Aβ, complement proteins, NOGO-A) increases during life and plateaus at high levels in old age, further amplifying the negative effect of PIRB/LILRB2 activation on synaptic integrity. When the expression of ligands is detected, but the role in plasticity and aging via PIRB/LILRB2 is still unresolved, a ‘?’ next to the ligand denotes this ambiguity.

In the elderly, cognitive decline is associated with both a reduced capacity to form new synapses and an intensified elimination of existing synapses. Supporting this notion, recent research indicates that the aging human brain exhibits larger, more stable excitatory synaptic structures, whereas younger brains feature smaller and more abundant plastic synapses [78]. Ig-like receptors are expressed in neurons and play a crucial role in the neuronal cascade responsible for synaptic weakening, activity-dependent pruning, and synaptic elimination [16,33,34,79]. In healthy brains, these receptors facilitate synaptic pruning during activity-dependent stages of brain development and contribute to the essential weakening of synapses in learning and memory mechanisms in adults [33,34]. However, with aging, they are ‘commandeered’ by high-affinity ligands associated with neuroinflammatory, neurodegenerative, and immunosenescent conditions. As a result, their normal function in synaptic weakening and pruning, believed to depend on low-affinity ligands (MHC-I), shifts toward pathological synaptic elimination due to overstimulation with high-affinity ligands (Figure 1 and Table 1).

**Table 1:** Immune receptors in the nervous system: cell expression and function

Immune receptor	Cellular expression in nervous system	Function in pathological conditions	Ligands	References
**LILRBs PIRB**	Neurons, endothelial cells	Modulating threshold for excitatory synapse plasticity; synapse weakening and elimination	MHC-I, NOGO-A, Aβ, C4d, ANGPTL, OMgp, MAG, MOG, MPZ	[3,16,30,33,35,82,89]
**KIR**	Neurons?	?	MHC-I	[45]
**Ly49**	Neurons?	?	MHC-I	[122]
**TCRs (CD3ζ)**	Neurons	Modulating threshold for excitatory synapse plasticity	MHC-I	[12]
**CR3**	Microglia, astrocytes	Engulfment of synaptic material in early stages of AD; opposes release of tPA from microglia needed for Aβ degradation	iC3b	[68,73]
**C3aR1**	Neurons, microglia, endothelial cells	Promote proinflammatory secretome in microglia and astrocytes; neurite dystrophy; vascular inflammation and BBB permeability	C3a	[64,143,144]
**C5aR1**	Microglia, astrocytes	Promote proinflammatory secretome in microglia and astrocytes	C5a	[66,67,136]
**C5aR2**	Neurons	Promote neurite distrophy	C5a	[138]
**TLR**[1-10]	Microglia, astrocytes, neurons, NPCs, oligodendrocytes	Autophagy or release of pro-inflammatory cytokines	DAMPs, PAMPs, Aβ	[9,20,40,100]
**TLR2**	Microglia	Release of proinflammatory cytokines (AD, ALS); infiltrating perivascular phagocytes (ALS)	Aβ, LTA	[41,43,169,173,176,181,182]
**TLR4**	Microglia, astrocytes, nociceptors	Release of proinflammatory cytokines (AD, PD, ALS, stroke); blocks anti-inflammatory action of microglial TREM2 (AD); production of NLRP3 inflammasome (PD); HMGB1-induced activation in stroke; modulation of allodynia (AD)	Aβ, α-synuclein, HMGB1, HSP, LPS, Gal-3	[41,42,74,75,76,158,168,173,174, 175,185,196]
**TLR5**	Microglia	Release of proinflammatory cytokines (AD)	Aβ, HMGB1, HSP, flagellin	[166]

## Leukocyte immunoglobulin-like receptors (LILRs)

Leukocyte immunoglobulin-like receptors (LILRs), located in the neuronal compartment of the central nervous system (CNS), play crucial roles in various functions from axonal guidance in early development to age-related and pathological changes affecting synaptic strength [1,3,16,30,80]. The LILR genes are part of the leukocyte receptor complex region on human chromosome 19, which contains several receptors related to the immunoglobulin (Ig) superfamily, including killer Ig-like receptors [81].

In humans, the LILR family comprises 11 functional genes, including five activating (LILRA1, 2, 4–6), five inhibitory (LILRB1–5), and one soluble form (LILRA3) [7]. Activating receptors contain immunoreceptor tyrosine-based activating motif (ITAM), while inhibitory receptors carry immunoreceptor tyrosine-based inhibitory motif (ITIM); ITIMs recruit SH2-domain-containing phosphatases, which inhibit immune cell activation [82]. *LILR*A3 and *LILR*A6 exhibit copy-number variations, while the rest are conserved among individuals [83,84]. The LILR genes are showing large interspecies differences as they are rapidly evolving [85], making function analysis challenging using animal models. In rhesus macaques, there are eight LILR orthologs, five activating and three inhibiting receptors [86]. In mice, there are approximately eight genes coding for activating receptors (*Pira*1-8), and only one gene codes for an inhibitory receptor (PIRB), which is the most likely murine homolog of human LILRB2 [7]. In the immune system, human LILRB2 is expressed on myeloid cells and hematopoietic stem cells [87–89]. In mice, PIRB has a wider expression in the immune system, and is found on the surface of B-cells, granulocytes, macrophages, and mast cells [8,23,82]. Paired expression of activating and inhibitory receptors on immune cells is responsible for self- versus non-self-epitope recognition [82,90].

### Function and signaling

The function of LILRB2 in the brain has been particularly well explored via its mouse homologue, PIRB [1,3,16,33,52,80]. In mouse brains, PIRB is found in neurons in cerebral cortex, hippocampus, cerebellum, olfactory bulbs, retina [1,30,33,34,44,52,91–96]. PIRB protein levels, detectable from postnatal day 5 (P5) into adulthood [1], increase with aging [97] and in response to stroke [44,91]. PIRB protein is enriched in synaptosome preparations, suggesting it functions in a synaptic locale [1].

Furthermore, in line with its synaptic localization, PIRB’s synaptic functions have been consistently demonstrated. In a range of experiments using germline and neuron-specific genetic deletions of *Pirb*, as well as pharmacological blockade of the receptor, it was found that PIRB constrains excessive excitatory synapse strengthening, and negatively affects stability and density of dendritic spines [16,33,34,52]. Congruent with its synaptic effects, PIRB also negatively regulates learning and memory [1,3,16,30,33,34,44,52,79]. From within the postsynaptic side of excitatory synapses, PIRB maintains the threshold for NMDA receptor-dependent synaptic plasticity in a manner that allows the synapse to undergo not just strengthening but also activity-dependent weakening; this is observed as a presence of both synaptic long-term potentiation (LTP) and long-term depression (LTD) [3,16,31,33,52]. Without PIRB, only LTP is detected [16,33,52]. PIRB engages NMDAR signaling to promote retrograde release of endocannabinoids (eCB), which in turn decrease presynaptic glutamate release probability in activity-dependent manner [33,34]. PIRB is also found to negatively affect density and stability of dendritic spines, structures that house postsynaptic excitatory machinery [16,34,79]. Recent evidence suggests that PIRB engages non-ionotropic NMDAR function to promote downstream cofilin dephosphorylation and actin disassembly, thus leading to spine shrinkage concurrently with LTD [52,98]. Finally, consistent with negative regulation of synaptic strength and spine stability, PIRB also negatively influences learning and memory as seen in behavioral learning tasks, like delayed matching to place task, reaching task, and rotarod [33,44,52].

### Ligands and implications for aging and neurodegeneration

It is assumed that normative function of LILRB2/PIRB in the brain depends, at least in part, on low affinity binding of its cognate ligands, MHC-I molecules (*K*_d_ = 1.9–5.6 × 10^−7^ M) [8]. Experiments on various MHC class I mutant mice suggest that the interaction between MHC-I and PIRB at synapses activates downstream PIRB signaling, leading to synaptic pruning in healthy developing and adult brains [1,12,44,51,54]. For example, effects of *Mhc-I* and *Pirb* genetic deletions on ocular-dominance plasticity are phenocopies of each other [1,12,54,79], both have similar effect on LTP and LTD threshold, and synaptic pruning [51,55,99]. Moreover, soluble PIRB-AP construct binds endogenous neuronal MHC-I on L5 pyramidal neurons with a *K*_d_ similar to that measured in the immune system [1,82].

Microglia become senescent with age and, together with astrocytes [9,100], they increase the release of pro-inflammatory cytokines, like IFN-γ and TNF-α [9,101]. This results in increase in MHC-I expression in both astrocytes and neurons [101,102], tracking with an overall increase of MHC-I in aged brain [103]. Therefore, aging and inflammatory context could exacerbate LILRB2/PIRB-dependent synaptic elimination just by increasing the amount of its cognate ligand MHC-I.

However, there is a number of non-MHC ligands in the brain that bind to LILRB2/PIRB [3,30,31]. In the context of aging, binding of oligomeric amyloid-β (Aβ), a soluble synaptotoxic and neurotoxic protein species, is potentially of great consequence [3,104]. Binding of oligomeric Aβ to PIRB and LILRB2 is saturable, with *K*_d_ of ∼180 nM; this makes Aβ-PIRB interaction >10-fold stronger that MHC-I-PIRB interaction [3,104]. Functional significance of oligomeric Aβ-PIRB interaction has been demonstrated in animal and *in vitro* models of AD [3]. In APP/PS1 transgenic mouse model of AD, diminished performance on novel object test is rescued back to normal when mice are also null for *Pirb* [3]. In acute model of AD, when oligomeric Aβ is superfused over acute hippocampal slice preparation from WT brains, LTP is abolished; however, hippocampal slices from *Pirb-/-* mice exhibit normal LTP, suggesting that lack of PIRB confers functional resistance in the presence of oligomeric Aβ [3]. The first two Ig-like domains (D1D2) of PIRB and LILRB2 are identified as a binding site for oligomeric Aβ [3,104]. Small molecule inhibitors, identified through structure-guided selection, prevented binding of oligomeric Aβ to D1D2 of LILRB2, and reduced Aβ cytotoxicity *in vitro* [104]. Together, evidence so far suggests that LILRB2/PIRB receptor is one of the neuronal effectors of synaptotoxic and neurotoxic soluble oligomeric Aβ [3,104].

Other known ligands for LILRB2/PIRB are myelin-associated proteins NOGO-A, OMgp (oligodendrocyte myelin glycoprotein), MAG (myelin-associated glycoprotein), MOG (myelin oligodendrocyte glycoprotein), and MPZ (myelin protein zero) [30,35]. During development, *in vitro* and *in vivo* assays suggest that NOGO-A, OMgp, and MAG oppose axonal guidance, extension, and regeneration via PIRB [30,105] (Figure 1 and Table 1). In adult, OMgp–PIRB interaction has been implicated in regulating threshold for LTP in hippocampal synapses [31]. NOGO-A itself has been implicated in negatively regulating synaptic turnover and learning in adult animals [106,107], and inhibition of NOGO-A signaling rescues LTP in aged APP/PS1 mouse model of AD [108]. In addition, an endogenous antagonist of NOGO-A–PIRB interaction, lateral olfactory tract usher substance (LOTUS), was discovered [109,110]: LOTUS rescues axonal outgrowth *in vitro* in a NOGO-A–PIRB-dependent manner [110]. However, it appears that LOTUS also inhibits Aβ–PIRB interaction, reversing excessive spine loss seen in primary cortical neuronal cultures in the presence of Aβ [109]. However, additional work would be necessary to establish whether and how NOGO-A, OMgp, MAG, and LOTUS, affect LILRB2/PIRB function *in vivo* in the context of aging and AD (Figure 1). It is of note that disease-associated oligodendrocytes (DOLs) are found near amyloid plaques in AD, potentially ‘concentrating’ myelin-associated ligands of PIRB in the area of high synapse loss [111]. Moreover, transcriptome of DOLs in various neurodegenerative diseases, including AD, is characterized by increased expression of *MHC-I*, β*2M*, and complement component *C4* (see the next paragraph); these are all genes coding for ligands of LILRB2/PIRB [111,112]. *C4* gene expression in the brain is up-regulated in an age-dependent and region-dependent manner, with corpus callosum showing the fastest ‘aging’ in terms of its immune transcriptome [111,113].

Aging and inflammation lead to increased expression of complement component C4 throughout cortex [97]. C4 is a part of the classical complement cascade; it undergoes a series of cleavage events, generating opsonins, and eventually terminal cleavage product, C4d. *In vitro* binding assays demonstrated saturable high-affinity binding of C4d for both LILRB2 and PIRB at ∼0.63 μM [112]. It is known that C4d increase is pronounced in the context of AD [114]. However, it remains to be seen if C4d–PIRB interaction results in synapse elimination in aged mice and mouse models of AD.

In addition to aging and AD leading to increase in protein levels of both LILRB2 receptor [97] and its ligands [97,103], many pathological conditions further exacerbate this process, raising the probability of excessive excitatory synaptic weakening and removal via LILRB2 [1,3,80,91,95,96,111,115–118] (Figure 1). In MCAO mouse model of stroke, the expression of PIRB and its MHC-I ligands (H2-K and H2-D), go up and peak at around 7-day post-reperfusion [44,91]; the elevation in all three proteins appears to be neuronal [91], suggesting that previously described role for MHC-I and PIRB in promoting LTD [12,16,33,52] can contribute to the pathological synapse loss in the ischemic penumbra around the infarct zone. Genetic removal, or acute blockade of PIRB speeds up the motor recovery post-MCAO [44,91]. Epileptic seizures, one of the co-morbidities of AD, also raise levels of neuronal and astrocytic LILRB2 and PIRB [115]; in pilocarpine-induced epilepsy model, PIRB levels go up more than 2-fold, peak at 7 days post seizure but remain at stably elevated level for at least 60 days [115]. Sleep-related disorders are another set of age-related co-morbidities; in chronic sleep restriction mouse model, up-regulation of hippocampal PIRB coincides with synaptic loss and cognitive impairments; knockdown of neuronal PIRB or blockade of downstream ROCK2 (Rho associated coiled-coil containing protein kinase 2) with fasudil alleviates synaptic dysfunction and cognitive impairments [118]. Diabetes Type 2, an age-related metabolic disorder, is also characterized by cognitive problems and synapse injury. In leptin receptor-deficiency mouse model of diabetes, synapses are smaller and less numerous, and performance on Morris Water Maze impaired [117]. In this mouse model, as well as *in vitro* neuronal cultures exposed to high glucose treatment, PIRB protein levels are elevated [117]; knockdown of PIRB with lentiviral shRNA rescued synaptic density in hippocampus, pyramidal neuron dendritic complexity, and Morris Water Maze performance [117].

Considering the implication of PIRB in various CNS pathologies associated with aging, there is an interest in therapeutic targeting of this receptor. A proof-of-concept experiment that demonstrated the benefit of acute PIRB blockade on synaptic function was application of soluble extracellular domain of PIRB (sPIRB) into visual cortex of adult mice affected by amblyopia (cortical blindness) [79]. Soluble sPIRB acted as a decoy receptor and caused endogenous PIRB to stop signaling. The outcome of this experiment was an increase in the number of dendritic spines in affected visual cortex via *de novo* formation of spines, and rescue of visual acuity toward normal levels [79]. In the context of stroke, delivery of sPIRB as well as anti-PIRB antibody into the affected hemisphere resulted in smaller infarct areas and faster motor recovery [91]. Couple of groups identified small molecules or peptides that interfere with binding of oligomeric Aβ to PIRB [92,104]; in *in vitro* assays these reagents rescued neurite and synapse loss, and reversed neurotoxic effect of Aβ [92,104]. Collectively, these experiments demonstrate the therapeutic potential of targeting LILRB/PIRB to promote the *de novo* emergence of synapses, synaptic survival in pathological conditions, and the restoration of correct neural circuit function even in adult and aging brains.

Finally, LILRB2 and PIRB also function as binding and internalization receptors for reovirus serotype T3, which shows CNS neuronal tropism [119]. Given that viruses can directly bind to this and other immune receptors (e.g., TLRs [119]) and are a known risk factor for some neurodegenerative diseases [120,121], understanding in greater detail the interaction between external pathogens and internal DAMP-like molecule activation of receptors becomes a topic of interest.

## Other related receptors in the brain

The Killer cell immunoglobulin-like receptor (KIR) genes, akin to LILRBs, belong to the Ig superfamily and feature intracellular ITIM domains. These genes are transcribed in brain regions associated with synaptic plasticity and neurogenesis [45].

The Ly49 family of receptors differs from the Ig-like domains found in LILRBs, featuring a lectin-binding domain in the extracellular portion but maintaining inhibitory ITIM domains intracellularly. Notably, the main ligands for this receptor family are MHC-I molecules [122]. Transcripts of Ly49 receptors are present in the adult brain, notably in sensory cortical areas (V1 and V2), auditory areas (A1 and AUD), all layers of the hippocampus (DG, CA3, and CA1), and in pathways connecting various cortical regions such as the retrosplenial cortex, presubiculum, and subiculum [122].

CD3ζ (CD3 zeta) serves as an adapter molecule for T-cell receptors (TCR), belonging to the Ig superfamily [46]. Notably, CD3ζ lacks an extracellular binding domain, featuring only intracellular signaling domains [46]. However, expression studies and functional work on CD3ζ in adult mouse brains could be used as a proxy for TCR complex function [12]. Mice lacking CD3ζ exhibit enhanced hippocampal CA3-CA1 LTP and abrogated LTD, mimicking the phenotype observed in other MHC-I null mice [12,99]. These findings collectively suggest a role for CD3ζ, and consequently, the TCR complex, in the negative regulation of synaptic plasticity in adults.

The role of KIR, Ly49, and CD3ζ in the aging brain has not been extensively investigated to date. Nevertheless, considering that neuroinflammation leads to increased levels of MHC-I expression [101,123], it is plausible that signaling through these receptors may be enhanced in the aging brain.

## Complement receptors mediate early synaptic removal in the aging brain and promote neuroinflammation

The complement system, an integral part of the innate immune system, plays a crucial role in safeguarding against invading pathogens. Moreover, it is intricately involved in diverse functions within the brain, encompassing developmental processes, neuroinflammation, and neurodegeneration [58,60,124]. Comprising three distinct cascades—classical, lectin, and alternative—the complement system involves cognate ligand–receptor pairs. Complement components, along with complement receptors, are distributed across various brain cell types. They can be induced in resident neurons, microglia, astrocytes, endothelial cells, oligodendrocytes, and cerebrovascular smooth muscle cells [64,124].

A well-established role of the complement system in the brain unfolds during development, particularly when the classical complement cascade recruits microglia in an activity-dependent manner. This activation orchestrates the synaptic pruning of thalamic areas, underscoring its pivotal developmental significance [2,21,125]. C1q, an initiating molecule in the classical cascade activates a downstream component C3. Both C1q and C3 are found at retinogeniculate synapses; C3, via C3 receptor (CR3) expressed on microglia, promotes microglial engulfment and removal of presynaptic terminals [2,21,125]. In addition to retinogeniculate synapses, there is evidence of complement-dependent pruning in cerebral cortex, as *C1q-/-* mice have excess axonal boutons and lower threshold for seizures [126].

**Figure 2:**
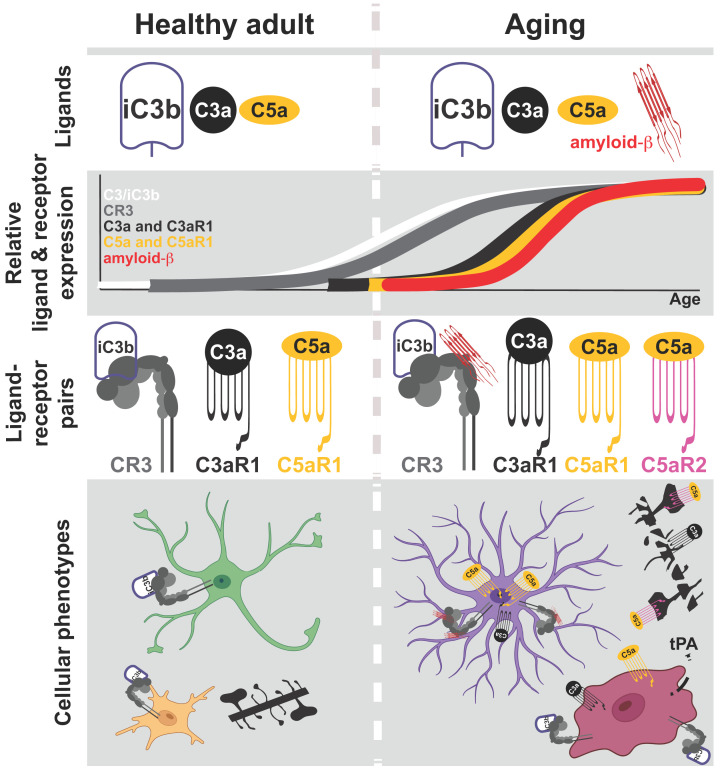
Complement receptor function changes with aging Top panel: Illustration of cognate complement ligands (opsonin iC3b, anaphylatoxins C3a and C5a) present in the brain of healthy adults (left) and in aging individuals (right). Note the availability of amyloid-β as a ligand in aging. iC3b and amyloid-β bind to CR3, while C3a and C5a bind to C3aR1 and C5aR1/C5aR2, respectively. Second panel from the top*:* Depiction of the relative amounts of complement receptors and ligands continuously throughout healthy adulthood and aging. *Third panel:* Illustration of different ligand-receptor pairs corresponding to the periods of healthy adult and aging. Bottom panel: As complement ligand–receptor pairs change or become more abundant with aging, cellular phenotypes for neurons, microglia, and astrocytes change as well. In healthy adults, neurons exhibit normal synaptic densities (black dendrite), astrocytes are neurotrophic (green cell), and microglia go between surveilling and activated (yellow cell) with an anti-inflammatory secretome. However, with aging, amyloid-β-CR3 signaling results in reactive astrocytes (purple cell) that overproduce C3. iC3b is generated and, together with amyloid-β, overactivates microglia (dark red cell), adopting an amoeboid shape and pro-inflammatory secretome. Excess C3 also results in high concentrations of anaphylatoxins C3a and C5a, which promote further pro-inflammatory phenotypes of both glial cells by binding to C3aR1 and C5aR1. C3a and C5a also bind to neurons via C3aR1 and C5aR2, coinciding with dystrophic neurites.

As the aging process unfolds, complement receptors play a role in instigating early synaptic loss observed in various neurodegenerative conditions. Furthermore, they contribute to neuroinflammation, intensifying the symptoms of AD, amyotrophic lateral sclerosis (ALS), stroke, traumatic brain injury (TBI), and epilepsy [124]. Recent studies have implicated complement receptors such as CR3 (receptor for a C3-cleavage product iC3b), C3aR (receptor for anaphylatoxin C3a, also a cleavage product of C3), and C5aR (receptor for anaphylatoxin C5a, a cleavage product of C5) in the early synapse loss observed in AD. These receptors also play a key role in propagating neuroinflammation by activating microglia and astrocytes. Additionally, they contribute to altering blood–brain barrier (BBB) permeability, facilitating peripheral lymphocyte infiltration [64,66] (Figure 2 and Table 1). It is worth stressing that these receptors are common to all three complement cascades, and therefore various pathogens and inflammatory molecules can contribute to neuroinflammation via complement system.

## Complement receptor CR3

Complement receptor 3 (CR3) has been associated with early synapse loss in the J20 mouse model of AD via activation of the classical complement cascade [68]. In J20, an initiating component of the classical complement cascade, C1q, is found to be associated with synapses as early as one month of age, well before obvious plaque deposition [127]. Moreover, it was found that C1q deposition on synapses was needed for the synaptotoxic effect of oligomeric Aβ: blockade of C1q with a function-blocking antibody rescued hippocampal LTP to levels seen in healthy animals of the same age. Finally, synaptic material tagged with cleavage products of C3 was engulfed by microglia in a CR3-dependent manner upon exposure to oligomeric Aβ [68]. Inhibiting C1q, or downstream component C3, or its receptor CR3 resulted in a decreased number of phagocytic microglia, alleviating early synapse loss. This work by Hong et al. [68] clearly demonstrated that components of classical complement system, known to be engaged in developmental synaptic pruning, can be inappropriately activated later in life and contribute to early synapse loss in the context of AD.

Unexpectedly, microglial CR3 has been found to play a role in regulating levels of Aβ [73]. Using *in vitro* approach in cultured microglia, it was found that deletion of *Cr3* gene promotes release of enzymatic factors from microglia, including tissue plasminogen activator (tPA), that degrade extracellular Aβ into non-synaptotoxic and non-neurotoxic species [73] (Figure 2 and Table 1). *Cr3* deletion in APP transgenic mouse model resulted in reduced plaque deposition even though the expression of amyloid precursor protein (APP) and its processing into Aβ remained unaffected. This points to an involvement of CR3 in enzymatic digestion of extracellular Aβ. Small-molecule inhibitor of CR3, LA-1, increased digestion of Aβ *in vitro* and lowered Aβ levels *in vivo* [73]. Collectively, two distinct and opposing functions of CR3 - promoting microglial phagocytosis of amyloid and inhibiting enzymatic clearance of Aβ - are implicated in the AD process. Whether microglial senescence and/or increased pro-inflammatory context influences in which direction CR3 functions remains to be seen.

## Complement receptor CR1

In genome-wide association studies (GWAS), complement receptor CR1 exhibits a significant association with AD [69]. CR1 binds cleavage products C4b, a complex of C3b/C4b products, and a complex of C1q/MBL (mannose-binding lectin). CR1 is detected in microglia and astrocytes within the human brain *in situ* [128,129]. Single-nucleotide polymorphisms (SNPs) in CR1, identified through GWAS, alter the expression levels of astrocytic CR1 and its binding affinity with ligands [129]. Nevertheless, the presence of these SNPs does not predict the occurrence of AD, nor does it correlate with various AD endophenotypes such as memory impairment, total tau, Aβ1-42, or tau phosphorylated at threonine 181 levels [129,130]. Given the strong association between CR1 and AD, influence of peripheral CR1 has to be considered.

## Anaphylatoxin receptors promote neuroinflammation

Apart from the direct opsonization and removal of synapses by microglia, the complement cascade plays a crucial role in promoting an inflammatory response. Cleavage within the complement cascade results in the generation of two anaphylatoxins, C3a and C5a, which contribute to inflammation and activation of target cells by binding to their respective cognate receptors—C3aR1, C5aR1 (CD88), and C5aR2 (C5L2). Upon activation, these G-protein coupled receptors induce chemotaxis and cellular activation, including the production of cytokines [131,132] (Figure 2 and Table 1).

### C5aR1 and C5aR2

Aβ has been shown to directly activate both the classical and alternative complement pathways [133–135], resulting in downstream generation of C5a after a series of cleavages. Upon binding to C5aR1 (CD88) expressed on microglia and astrocytes, C5a anaphylatoxin activates both cell types in a C5aR1-dependent manner [136]. Antagonization of C5aR1 signaling has been demonstrated as beneficial in mouse models of AD. Administering a C5aR1 antagonist (cyclic hexapeptide, PMX205) orally for 2–3 months led to a ∼55% reduction in fibrillar and total amyloid deposits in the 12- to 15-month-old Tg2576 mouse model of AD; CD45 and GFAP-reactive areas were also decreased, suggesting fewer activated microglia and astrocytes in this mouse model [66]. Treating the 3xTg mouse model of AD with PMX205 at ∼20 months of age resulted in a ∼70% decrease in hyperphosphorylated Tau. In addition, lower thioflavin staining for plaques and a minor effect on CD45 microglia immunoreactivity were also observed [66]. In Tg2576 mouse model, PMX205 preserved the integrity of presynaptic terminals in stratum lucidum of CA3 field, suggesting that lower inflammation after chronic blockade of C5aR1 also protects presynaptic terminal from pathological removal. Consistent with the synapse preservation with PMX205, Tg2576 mice have rescued performance on passive-avoidance task [66]. In a separate study, a C5ar1 knockout mouse was crossed with a mouse line carrying the human Arctic AD mutation [65]. Genetic ablation of *C5ar1* preserved memory and maintained neuronal complexity in the CA1 region of the hippocampus; it also increased degradation and clearance pathways in microglia at the expense of induction of inflammatory genes, and shifted microglial cells toward a less inflammatory phenotype [65] (Figure 2).

Conversely, intermittent stimulation of C5aR1 with an agonist, EP67, provided protection against the effects of amyloid protein by enhancing the phagocytic activity of microglia [137]. In 5xFAD mice, intermittent oral treatment with EP67 at 3 months of age resulted in a significant reduction of both fibrillar and non-fibrillar Aβ, reduced astrocytosis, preserved synaptic and neuronal markers, and memory function [137]. This experiment suggests that the degree and duration of C5aR1 receptor activation can induce distinct microglial responses. Early and phasic stimulation promotes beneficial phagocytic behavior, while chronic exposure to anaphylatoxin in the context of prolonged neuroinflammation shifts microglia toward an inflammatory phenotype, further propagating neuroinflammation (Figure 2). This dynamic is also reminiscent of how the degree of activation of TLRs influences microglial behavior, with low-grade stimuli coinciding with microglial phagocytosis and clearance of cytotoxic Aβ.

Besides C5aR1, another receptor for C5a, C5aR2, is also present in the brain [138]. In human AD cohorts, antibody staining against both C5aR1 and C5aR2 indicated elevated immunoreactivity relative to controls and vascular dementia (VD) patients. C5aR2-associated signal is found in neurons in control subjects, and in AD samples it overlaps with neurofibrillary tangles. Around plaques, C5aR2 associates with dystrophic neurites and neurofibrillary tangles. Moreover, signals for both C5aR1 and C5aR2 colocalize with hyperphosphorylated tau [138]. Nevertheless, a recent mouse study suggests that the activation of C5aR2 can also exert a neuroprotective effect [67]. In the context of the Arctic model of AD, overexpression of C5a anaphylatoxin, while overall detrimental, delayed the upregulation of some AD-, complement-, and astrocyte-associated genes that are normally elevated in AD, suggesting a concomitant anti-inflammatory process [67].

### C3aR1

The presence of Aβ activates astrocytes, leading to increased production and release of the C3 protein, which aligns with the elevated levels of C3 observed in brain tissue from AD patients and APP transgenic mice [139,140]. Aβ-dependent C3 release from astrocytes is likely via Aβ–TLR–NFκB astrocytic cascade [9]. Increase in astrocytic C3 leads to excess C3a, which binds to C3aR1 receptors expressed on neurons and microglia [141,142]. Neuronal C3a–C3aR1 interaction leads to disrupted dendritic morphology and network function [142]. At the same time, upregulation of astrocytic C3 will also result in activation of microglial C3aR1 [141]; acute exposure to Aβ can lead to microglial phagocytosis, but, in the condition of chronic exposure to C3/C3a, microglial phagocytosis is attenuated [141].

Furthermore, the activation of C3aR1 is correlated with tau pathology, consistent with the observation that levels of C3aR1 positively correlate with Braak staging [143]. Inhibition of C3aR1 in PS19 mice rescues tau pathology, attenuates synaptic loss, neuroinflammation and neurodegeneration [143]. C3aR1-dependent transcription factor network includes STAT3 known to mediate tau pathology; this network also includes other genes related to late-onset AD [143]. All together, these studies, indicate that neuronal (over)production of Aβ triggers a complement-dependent intercellular cross-talk in which Aβ activates astrocytic NFκB that in turn potentiates secretion of C3. This promotes a vicious cycle by which increased C3 leads to higher concentration of C3a anaphylatoxin, which in turn interacts with neuronal and microglial C3aR1 to impair microglial Aβ phagocytosis and promote tau pathology.

Beyond neurons and microglia, C3aR1 is present on the endothelial cells of blood vessels [64]. Immune and vascular dysfunction are implicated in aging and AD, and C3aR1 found n endothelial cells appears to increase age-related risk for dementia and AD by promoting vascular inflammation and disrupting barrier integrity [64]. C3a–C3aR1 signaling in endothelial cells is responsible for robust age-dependent increase in vascular cell adhesion molecule 1 (VCAM1) and vascular inflammation, age-dependent peripheral lymphocyte infiltration, including CD8^+^ T-cell infiltration in aged cortex, thalamus, hippocampus, and caudate putamen, and increase in BBB permeability. Global and endothelial cell-specific deletion of C3aR1 attenuates all of these phenotypes. C3a-mediated barrier disruption is dependent on Ca^2+^ mobilization and alteration of VE-cadherin through cytoskeletal activation. In addition to changes in vasculature morphology and barrier integrity, endothelial C3aR1 contributes to age-related microglial activation and mild but significant decrease in cortical volume; both microglial activation and neurodegenerative phenotypes are rescued in *C3ar1-/-* mice [64].

Unsurprisingly, C3aR1 has also been implicated in recovery after injury, when neuroinflammatory environment contributes to injury-related pathology. C3aR1 has a detrimental impact on BBB permeability early in post-stroke recovery, yet exhibits a neuroprotective effect in the chronic phase of recovery [144]. Perioperative cognitive disorders are also mediated by C3 upregulation in astrocytes and C3aR1 in microglia of hippocampus [145]. The blockade of C3aR1 by C3aR antagonist (C3aRa) attenuates post-surgical neuroinflammation. Additionally, the post-operative dysfunction of the brain–CSF barrier at the choroid plexus is rescued, leading to an improvement in trace fear-conditioning performance [145]. Altogether, the body of work suggests that while some disorders initially involve peripherally derived complement components owing to a compromised BBB in stroke, TBI, and multiple sclerosis (MS), CNS-produced complement becomes the dominant chronic source of these components and drives local neuroinflammation in many neurodegenerative diseases.

Lastly, various innate immunity effector mechanisms can either synergize with or antagonize the complement system, tightly regulating the immune response to pathogens and misfolded proteins, such as Aβ and tau. In the periphery, C5aR1 was found to synergize with TLR2 and TLR4 to enhance proinflammatory cytokine responses (TNF-α and IL-1β) in *in vivo* mouse models, mouse macrophages, and human monocytes [146]. TLRs, Fc receptors, chemokines and other mediators are engaged by their own cognate ligands, and their signaling machinery can drive ‘inside-out’ activation of CR3, increasing CR3 affinity/avidity, thus controlling its immune and inflammatory functions [147].

In conclusion, presented findings indicate that targeting late components of the complement cascade might be a more promising therapeutic strategy compared to targeting C1q or C3 earlier in the cascade, given that these early components serve other beneficial functions [148–150]. Moreover, when contemplating pharmacological interventions targeting CR3, C5aR1, and C5aR2, it is crucial to take into account the ‘yin-yang’ aspect of their functions. This refers to the dual roles these receptors play, acting in both proinflammatory and anti-inflammatory capacities depending on specific conditions. Understanding this dynamic nature is essential for developing effective therapeutic strategies.

## Toll-like receptors: microglial overactivation in the aging brain and neurodegeneration

TLRs stand as the evolutionarily oldest innate immune receptors, with prototypical family members dating back to Cnidaria [27,151]. Vertebrate TLRs emerged about half a billion years ago, after a series of gene duplication, pseudogenization, purification, and positive selection events [27,28]. In spite of these extensive changes, TLRs in vertebrates have preserved the prototypical layout: extracellular domain consists of hydrophobic tandem leucine-rich repeat (LRR), which mediates the recognition of PAMPs and damage-associated molecular patterns (DAMPs). The most salient difference between invertebrate/early chordate versus vertebrate TLRs is the loss of multiple cysteine cluster LRRs in the extracellular domains; only single-cysteine-cluster TLRs are found in vertebrate species studied to date [27,152].

Humans possess ten identified members of the TLR family (*TLR1-10*), whereas mice have 12 different TLRs (*Tlr1–Tlr9* and *Tlr11–Tlr13*) [9,27]. Importantly, a subset of *TLR* genes is orthologous in all vertebrates (*TLR3*, *TLR4*, *TLR5*, and *TLR7*) [153], which facilitates interpretation of mouse studies in the context of human condition. The binding of PAMPs/DAMPs to the majority of human TLRs initiates a conserved TLR–NF-κB signaling cascade [25,154], leading to the release of pro-inflammatory cytokines [9]. In the brain, TLRs are mostly expressed not only in microglia [155,156] and astrocytes [155,157] but also in neurons [158], neural progenitor cells (NPC) [159] and oligodendrocytes [155].

### Function and signaling

Similar to complement receptors, TLR activation can lead to opposite functions: on one hand to autophagy [41,42], and on the other to the release of proinflammatory cytokines [43]. This difference is made by the degree of TLR activation [41,160], with overactivation resulting in the release of IFN-I and other proinflammatory cytokines (TNF-α, IL-6, IL-12; [161]).

All TLRs, except for TLR3, recruit myeloid differentiation factor 88 (MyD88) via their Toll/interleukin-1 receptor (TIR) domain; TLR1, 2, 4, and 6 also recruit additional adaptor molecules (e.g. CD14 and TIR domain containing adaptor protein – TIRAP) that connect TIR domain to MyD88 [32]. Recruitment of MyD88 by TLR in turn causes binding of Interleukin-1 receptor (IL-1R) associated kinase (IRAK) family of proteins to the complex, which causes phosphorylation of I-κB and release and translocation of the transcription factor NF-κB to the nucleus where it regulates expression of various proinflammatory cytokines [154].

### Ligands

Intracellular localization of TLR receptors correlates with the repertoire of its ligands. TLRs can be found within cells either on the plasma membrane (in human TLR1, 2, 4, 5, 6, 10) or in endosomes (TLR3, 7, 8, 9) [9]. Plasma-membrane TLRs bind various extracellular PAMPs (e.g. bacterial lipopolysaccharide – LPS, flagellin, lipoteichoic acid – LTA, diacyl and triacyl peptides, zymosan, influenza A virus); endosomal TLRs bind intracellular pathogen epitopes, including viral dsDNA and ssRNA, and bacterial CpG dinucleotides [9,162].

In the context of normal aging of the brain and neurodegeneration, the detection of various DAMPs by TLRs becomes particularly relevant. These DAMPs include misfolded and amyloid proteins, heat-shock proteins, and various alarmins [9,74,163–165]. To date, all of the TLRs have been shown to bind amyloid-β (Aβ) [9,41,42,74,163,166–170], save for TLR10 that recognizes solely influenza A virus [171]; this suggests that Aβ can trigger proinflammatory response of microglia and astrocytes in a TLR-dependent manner. The majority of TLRs, along with Aβ, show an increase with age in both mice [172] and humans [97], implying an enhanced impact of TLR-Aβ interaction in promoting age-related neuroinflammation. In AD patients, there is an additional marked increase in expression for the TLR2, TLR7, and CD14 (another LPS receptor) [24,161], further amplifying the effect of Aβ on glial activation via TLRs (Table 1).

**Figure 3:**
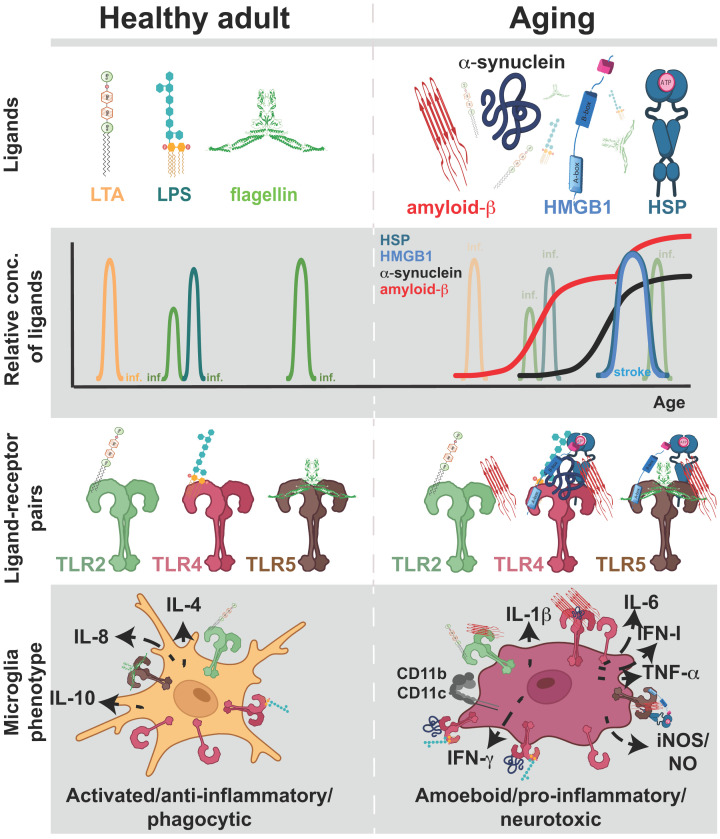
TLR activation and microglial phenotypes with aging Top panel: Illustration of some ‘classic’ TLR ligands of bacterial origin (LPS, LTA, flagellin) present during healthy adulthood (left) and in aging individuals (right). Amyloid-β, α-synuclein, HST (heat-shock proteins), and HMGB1 (high mobility group box 1) become available in aging. Second panel: Depiction of the relative amounts of various TLR ligands in healthy adulthood and aging. The presence of pathogen-related ligands is temporary due to the passing nature of most infections (‘inf’). Misfolded proteins and alarmins come to play prominent roles with aging. An increase in HMGB1 and HST is illustrated as temporary due to events like stroke (in blue letters); stroke can lead to a superimposing increase in amyloid-β. Third panel: Illustration of different ligand–receptor pairs corresponding to healthy adulthood and aging periods. Bottom panel: As TLR ligand–receptor pairs change or become more abundant with aging, microglial phenotypes change. In healthy adults, microglia get activated by TLR ligand–receptor binding due to passing infections, presenting an anti-inflammatory secretome, releasing interleukins IL-4, IL-8, IL-10, and exhibiting defensive phagocytic behavior. With aging, microglial TLRs are overactivated by binding amyloid-β, α-synuclein, HST, and HMGB1, in addition to ligands that increase with infection. Microglia adopt an amoeboid shape, exhibit a proinflammatory secretome (TNF-α, IL-6, IL-1β, NO/iNOS, IFN-γ, IFN-I), increase expression of CR3, increase expression of integrins CD11b and CD11c that are part of CR3 and C4 receptors, respectively, and become neurotoxic.

Functional studies in mouse models of AD or *in vitro* microglial cultures suggest that the degree of activation of TLRs by Aβ, or by concomitant PAMP/Aβ, can either oppose or promote neurodegenerative changes. Low-grade activation increases autophagy and clearance of Aβ oligomers [41,42,173,174], while over-activation causes microglial release of proinflammatory cytokines IL-6, INF-I, TNF-α among others [41,43,76,156,175], which aggravates dementia symptoms and propagates neurodegeneration (Figure 3 and Table 1).

## TLR2 and TLR5

Studies involving inhibiting the function of Toll-like receptor 2 (TLR2) strongly implicate its involvement in AD. Aβ1-42 peptides induce the expression of inducible NO synthase, proinflammatory cytokines (TNF-α, IL-1β, and IL-6), and integrin molecules (CD11b, CD11c, and CD68) in mouse primary microglia and BV-2 microglial cells in a TLR2-dependent manner [43] (Figure 3 and Table 1); *Tlr2-/-* derived cells are protected from Aβ1-42-induced release of proinflammatory cytokines [43]. In the 5XFAD mouse model of AD, selectively disrupting the interaction between TLR2 and MyD88 results in TLR2-specific inhibition of microglial activation induced by fibrillar Aβ1-42 and LTA. This is a TLR2-specific effect, as only the engineered peptide that mimics MyD88-binding loop of TLR2, but not other TLRs, has an effect. Intranasal delivery of the mimic-peptide reduced hippocampal glial activation, lowered Aβ burden, attenuated neuronal apoptosis, and improved memory and learning in 5XFAD mice [169]. These results are in line with reports that TLR2 goes up with age and in AD patients [24,97].

Nevertheless, studies exist that report an opposite effect of TLR2 activation [41,160,173,176]. Genetic deletion of TLR2 speeds up cognitive decline with normal aging, and supercharges it in the context of AD [160,176]. In addition, in early stages of AD when Aβ is increased but microglia are still in resting state, co-stimulation of TLR2 and TLR4 with PAMP ligands like LPS, MPL, and Pam3Cys, appears to promote microglia-mediated autophagy, as evidenced by lowering of Aβ deposits in the rat brain, and the rescue of spatial and working memory [41]. These seemingly conflicting findings could be reconciled by considering the age-related shift in microglial phenotypes. Aging study of microglia *in vitro* shows that age-associated responses by microglia include reduced phagocytosis and reduced migration, suggesting that microglia loses the capability to convert the TLR signals into autophagy as they age, resulting in poorer Aβ clearance from brain parenchyma later in life [156], and increased release in pro-inflammatory cytokines [43].

The TLR2-mediated cell response is also implicated in Parkinson’s disease (PD), another age-related neurodegenerative disorder. PS is characterized by intracellular deposition of α-synuclein known to promote neuronal degeneration [177]. Postmortem brains of PD patients exhibit increased proteins levels of TLR2 [177]. In addition, neuronal TLR2 activity has been shown to increase accumulation of endogenous α-synuclein in cultured neurons [178]. TLR2 activation also contributes to inflammatory process associated with ALS. In the post-mortem spinal cords of ALS patients, increase in *TLR2* mRNAs has been detected [179]. In addition, immunoreactivity for CD14, a co-receptor for TLR2, has been linked to infiltrating perivascular phagocytes in ALS spinal cords [24], a process not observed in AD or PD [180]. In stroke, most of the evidence suggests the involvement of TLR2 [164,181,182]. Transient focal ischemia increases levels of TLR2 mRNA; *Tlr2-/-* have reduced infarct size compared to the wild-type mice [182].

Apart from TLR2, Toll-like receptor 5 (TLR5) has also been implicated in amyloid plaque burden associated with AD. *In vivo* blockade of TLR5 with soluble decoy receptors (sTLR5Fc) significantly lowered the amyloid plaque burden and Aβ42 levels in TgCRND8 mouse model of AD. However, there was no behavioral rescue in the mice expressing sTLR5Fc [166].

## TLR4

### Neurodegenerative disease

Toll-like receptor 4 (TLR4), originally discovered in Drosophila ([183], has emerged as a key player in the etiology and progression of age-related neurodegenerative and inflammatory diseases [161]. In mouse models of AD and in post-mortem AD brains, TLR4 levels are increased [184]. A spontaneous loss-of-function mutation in the TLR4 gene markedly inhibits microglial and monocytic activation by aggregated Aβ, resulting in a significant reduction in the release of inflammatory products such as IL-6, TNF- α, and nitric oxide [184].

Moreover, TLR4 contributes to Aβ-induced inflammation by blocking the anti-inflammatory pathway mediated by microglial TREM2 (Triggering Receptors Expressed on Myeloid Cells 2) [185]. In APP/PS1 mice, both TLR4 and TREM2 are elevated at the protein level [185]; the treatment of these mice with LPS, a ligand for TLR4, resulted in worsened cognitive impairment in these mice, suggesting that superimposition of systemic inflammation due to bacterial infection could speed up the AD progression. Moreover, while TLR4 remained up-regulated upon LPS treatment, TREM2 was downregulated, suggesting that LPS–TLR4 interaction during bacterial infections could down-regulate the anti-inflammatory action of TREM2 [185]. Notably, the relevance of TLR4 in AD etiology is supported by findings in the Italian population, where the presence of the TLR4 Asp299Gly polymorphism appears to confer protection against late-onset AD [186].

Similar to TLR2, TLR4 has been implicated in promoting α-synuclein-dependent neuronal degeneration, as well as microglial and astrocytic activation in PD [177]. Postmortem examinations of brains from PD patients reveal an increased expression of TLR4 [177]. Moreover, when intracellular α-synuclein is released, it activates TLR4 on both microglia and astroglia leading to release of proinflammatory cytokines and reactive oxygen species, leading to further neuronal damage and worsening of disease symptoms in a TLR4-dependent manner [77] (Figure 3 and Table 1).

Aβ deposition is also implicated in PD, and the combined effects of Aβ and α-synuclein contribute to cognitive decline in these patients. Neurodegeneration and dementia in PD can be worsened by impairing the autophagy lysosomal pathway and protein clearance induced by neuroinflammation, and by increasing intracellular α-synuclein accumulation and aggregation [187]. TLR4 activation on microglia can counteract this process by enhancing the clearance of Aβ peptides and α-synuclein deposits in PD by enhancing microglia phagocytosis [77]. On the other hand, fibrillar α-synuclein and TLR4 are involved in promoting PD symptoms by inducing the NLRP3 inflammasome-mediated dopaminergic cell death [75,188]. Inhibition of NLRP3 activation by small-molecule inhibitor, MCC950, mitigated both motor dysfunction and dopaminergic neuron loss [188].

Similarly to TLR2, post-mortem spinal cords of ALS patients exhibit increase in TLR4 mRNA [179], and together with CD14 co-receptor, are linked to infiltrating perivascular phagocytes in ALS spinal cords [24,180]. Blocking antibodies for TLR2, TLR4, and CD14, attenuate extracellular superoxide dismutase type 1 (SOD1)-dependent microglia-mediated motor neuron injury [189]. In mouse models of ALS (hSOD1G93A), absence of TLR4, or its pharmacologic blockade with TAK-242, delays disease progression, attenuates glial reactivity in spinal cord, and reduces spinal motor neuron loss [190]. Like in AD, administering LPS to pre-symptomatic SOD1 mice activates TLR4, which in turn speeds up disease progression and death [179].

Furthermore, TLR4 is implicated in post-stroke inflammation [164,181,182]. Transient focal ischemia increases levels of TLR4 mRNA; *Tlr4-/-* have reduced infarct size compared with the wild-type mice [182]. During hemostasis, lack of oxygen and glucose leads to cellular damage and release of endogenous HMGB1 that activates TLR4, leading to immune cell infiltration, activation of microglia, expression of TNF-α and iNOS, and MMP-9 [191] (Figure 3 and Table 1). Intravenous injection of neutralizing anti-HMGB1 opposes these proinflammatory steps [191]. Notably, TLR4 polymorphisms have been linked to stroke. In ethnic Chinese in Taiwan, TLR4 C119A polymorphism is linked to stroke severity; Asp299Gly polymorphism implicated in protection from AD does not affect stroke severity [186,192].

### Peripheral involvement and pain regulation in aging

Beyond its central role in mediating inflammation through glial cells, TLR4 has been identified as a mediator of pain sensation in the peripheral nervous system [158,165]. TLR4 expression is observed in neuronal Nav1.8 nociceptor cells [158]. In a mouse model of neuropathic pain caused by spared nerve-injury (SNI), Nav1.8 nociceptors show increase in ATF3 expression and HMGB1 translocation from nucleus to cytosol [158]. These DAMP molecules in turn result in activation of TLR4 expressed in nociceptor cells, an event shown to be necessary for SNI-induced hyperalgesia; nociceptor-specific deletion of TLR4 results in higher pain withdrawal threshold, while nociceptor-specific recovery of TLR4 expression restores hyperalgesia [158]. Interestingly, the nociceptive TLR4-dependent mechanism appears to be specific to females [158]. In males, TLR4-positive immune cells [158] and microglia [165,193,194] appear to be involved in hyperalgesia, as these immune become more numerous in the DRGs on the injured side [158,165].

Aging and cognitive disability bring about alterations in pain processing mechanisms and the reporting of pain [195,196]. Individuals with dementia and AD often exhibit increased pain tolerance, resulting in heightened inflammation-induced tissue damage before the initiation of anti-inflammatory and analgesic therapy [195,196]. Conversely, chronic musculoskeletal pain experienced in midlife has been linked to an increased risk of depression and dementia later in life [197,198]. The interdependence of pain processing mechanisms, mood changes, and cognitive alterations has given rise to a new category known as nociplastic pain [195,199–202]. Recent research has elucidated a shift in the microglial TLR4-dependent mechanism for musculoskeletal pain sensation, providing insight into the attenuation of allodynia observed in AD [196]. In experimentally-induced pro-inflammatory arthritis in WT mice, pronociceptive TLR4^+^P2Y12^+^ microglia respond to neuronal release of Gal-3 (a DAMP ligand of TLR4) and ATP (ligand for the purinergic receptor P2Y12) by increasing the release of pro-inflammatory mediators, which in turn lead to nerve-ending sensitization. In the TASTPM mouse model of AD, a distinct subset of phagocytic TREM2+TLR4−P2Y12− microglia emerges, exhibiting an inability to bind either Gal3 or ATP. This microglial phenotype is associated with increased pain threshold, as measured by frequency of hind paw withdrawal to a mechanical stimulus, contributing to diminished pain sensation [196]. Therefore, the emergence of TLR4 negative microglia in the context of AD interferes with the TLR4-dependent mechanism of nociception, and could explain in part the attenuation of pain sensation in aged individuals and AD patients.

## Conclusions

**Figure 4:**
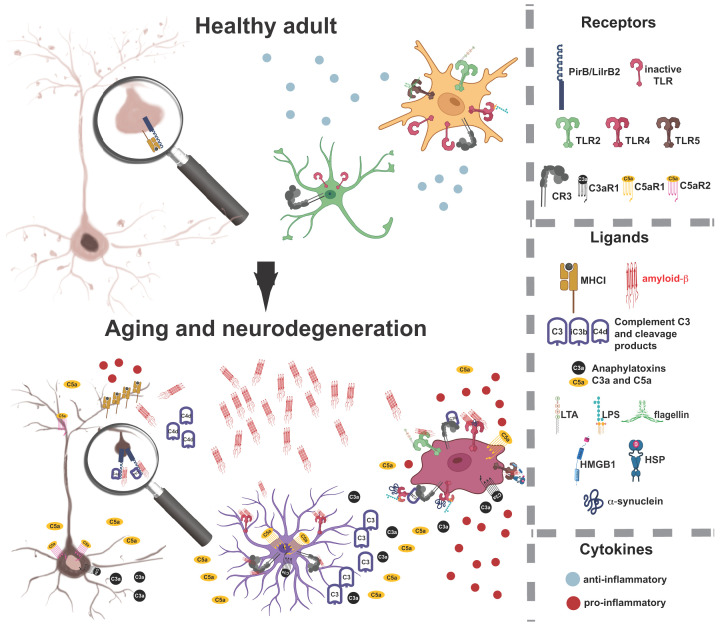
Immune receptors in aging brain orchestrate destructive intercellular communication Top panel: In the healthy brain of young adults, activated microglia (yellow) and astrocytes (green) release anti-inflammatory cytokines and engage in phagocytic removal of cellular debris when activated by DAMPs at low concentration. In neurons, PIRB/LILRB2 located in synapses bind major histocompatibility complex class I (MHC-I) and engage in the maintenance of synaptic capacity via a synaptic plasticity cascade that negatively regulates presynaptic glutamate release. Synaptic size distribution on a neuron (beige cell) reflects healthy control of synaptic strength; higher density of dendritic spines and presence of small plastic spines provides the capacity for new learning and memory storage. Bottom panel: In the aging brain and neurodegenerative conditions, a gradual increase in misfolded proteins (illustrated with amyloid-β) and various DAMPs produced by neurons activates the complement cascade, resulting in increased production of C4 by neurons, C3 by astrocytes, and downstream increase in C4d, C3a, C5a. Astrocytic C3 activates microglia via CR3. TLRs on both astrocytes and microglia are activated directly by Aβ and other DAMPs. In these conditions, activated astrocytes and microglia increase the production and release of proinflammatory cytokines. Activated microglia attains an amoeboid form (dark red cell), shifting astrocytes into a neurotoxic stage (purple cell). Pro-inflammatory cytokines like TNF-α and IFN-γ increase the production of neuronal MHC-I. In synapses, PIRB/LILRB2 now binds Aβ, C4d, and MHC-I, leading to excessive synaptic weakening, permanent dendritic spine removal, and synaptic loss. Anaphylatoxin receptors, C3aR1 and C5aR2 on neurons and C3aR1 and C5aR1 on microglia, bind their cognate ligands C3a and C5a, leading to neurite dystrophy on already stressed neurons (brown cell), while microglia increase the release of pro-inflammatory cytokines. This vicious cycle is additionally amplified as microglia become immunosenescent and lose its phagocytic capacity even for lower concentrations of DAMPs.

In the aging brain, immune receptors from Ig-superfamily, TLRs, and complement receptors promote synapse elimination, neurodegeneration, and neuroinflammation. Our proposed model delineates how cognitive decline associated with aging and neurodegenerative diseases arises from the coordinated activation of specific immune receptors—PIRB/LILRB2 and C5aR2 in neurons, TLRs and CR3 in astrocytes, and TLRs and CR3 in microglia (Figure 4). In a healthy state, the three-way intercellular communication is neurotrophic and surveilling, resulting in optimally maintained synaptic strength and well-preserved neuronal ramification. With aging, an excess of misfolded proteins and DAMP ligands bind to immune receptors expressed in neurons, propelling the cascade of excessive synapse loss. Simultaneously, misfolded proteins and DAMP ligands induce the overactivation of microglia and astrocytes, prompting these cells to transition into a proinflammatory and neurotoxic phenotype. This transition results in an additional up-regulation of immune receptor ligands, exacerbating synaptic loss and neurite degeneration via immune receptors located on the surface of neurons. Crucially, TLRs, complement receptors, and Ig-like receptors collectively play an essential role in driving the destructive cascade, as illustrated in Figure 4.

## Future perspectives

The intricate interplay between TLRs, Ig-like receptors, and components of the complement cascade could unveil the mechanistic link between infections and the heightened risk of neurodegeneration in later life. The well-established correlation between a history of viral infections and an increased incidence of cognitive problems and neurodegenerative disorders sets the stage for investigating the role of these immune receptors in mediating these effects (e.g. [120,203–206],).

Recent analyses of patient time series data from large databases such as FinnGen and UK Biobank have shed light on the intricate connection between viral exposure and the risk of neurological diseases later in life [206]. Notably, individuals exposed to at least 22 viruses exhibited an elevated risk of developing neurological diseases. For instance, viral encephalitis was associated with a 22–30 times higher likelihood of being diagnosed with AD, surpassing by 2-fold the risk conferred by APOE4, a known genetic risk factor [207]. Epstein–Barr virus was identified as a risk factor for MS, while influenza with pneumonia increased the risk for various neurodegenerative diseases, excluding MS [206]. Some viral infections were linked to an increased risk of neurodegenerative diseases up to 15 years later [206]. Viruses such as herpes zoster, herpes simplex 1/2, and human herpesvirus 6 were also identified as risk factors for neurological diseases in later life [120,203–206,208,209].

Despite the overwhelming evidence linking viral infections to accelerated cognitive decline, the precise mechanisms through which viral pathogens create a conducive environment for neurodegeneration remain incompletely understood. TLRs, Ig-like receptors, and complement cascade components emerge as plausible candidates for mediating the augmented risk of neurodegeneration, given that various pathogenic epitopes act as ligands for these receptors in the brain, akin to their role in the immune system [9,119]. Importantly, pathogen binding in the brain might trigger neuroinflammatory, synaptotoxic, and neurotoxic responses similar to the binding of endogenous ligands, misfolded proteins, and DAMPs.

In addition to increasing the risk of future neurodegenerative diseases, viral and bacterial infections may synergize with ongoing neurodegenerative processes through immune receptors in the brain, potentially worsening disease progression and symptoms. Given their significant involvement in promoting neurodegeneration and neuroinflammation, particularly in synergy with infections, immune receptors in the brain present a promising therapeutic target for combating age-related cognitive decline and neurodegenerative diseases. Future research in this area holds the potential to unravel the complex interactions between infections and immune receptors, paving the way for innovative therapeutic strategies to mitigate the impact of neurodegenerative disorders on cognitive health.

## Abbreviations

Aβ: amyloid-beta
AD: Alzheimer’s disease
ALS: Amyotrophic lateral sclerosis
β2M: beta2 microglobulin
BBB: blood–brain barrier
C1q: Complement protein 1q
C3aR: Complement receptor for anaphylatoxin C3a
C5aR: Complement receptor for anaphylatoxin C5a
CD: Cluster of differentiation
CR3: Complement C3 receptor
DAMP: damage-associated molecular pattern
GWAS: Genome-wide association study
HMGB1: High-mobility group box 1
IFN: Interferon
Ig: Immunoglobulin
IgSF: Immunoglobulin superfamily
IL: Interleukin
iNOS: Inducible nitrous oxide synthase
IRAK: IL-1R associated kinase
IRF3: Interferon regulatory factor 3
ITAM: Immunoreceptor tyrosine-based activating motif
ITIM: Immunoreceptor tyrosine-based inhibitory motif
LILR: leukocyte immunoglobulin-like receptors (A or B)
LPS: lipopolysaccharide
LRR: leucine-rich repeat
LTA: Lipoteichoic acid
MAC: Membrane attack complex
MHC-I: major histocompatibility complex class I
MHC-II: major histocompatibility complex class II
MMP-9: Matrix metalloproteinase-9
MyD88: Myeloid differentiation factor 88
NPC: Neural progenitor cell
PAMP: Pathogen-associated molecular patterns
PD: Parkinson’s disease
PIR: Paired immunoglobulin-like receptor
PRR: Pattern-recognition receptor
SH2: Src-homology 2
SNI: spared nerve-injury
SOD1: superoxide dismutase type 1
TCR: T-cell receptor
TIR: Toll/interleukin-1 receptor
TLR: Toll-like receptor
TNF-α: Tumor necrosis factor-alpha
tPA: Tissue plasminogen activator
TREM2: Triggering receptors expressed on myeloid cells 2
